# Comparative Effectiveness for Glycemic Control in Older Adults with Diabetes

**DOI:** 10.1007/s13670-017-0215-z

**Published:** 2017-07-28

**Authors:** Michael Quartuccio, Brian Buta, Rita Rastogi Kalyani

**Affiliations:** 1 Division of Endocrinology, Diabetes and Metabolism, Department of Medicine, Johns Hopkins University School of Medicine, 1830 East Monument Street, Suite 333, Baltimore, MD 21287 USA; 2 Department of Medicine, Johns Hopkins University, Baltimore, MD USA; 3 Center on Aging and Health, Johns Hopkins Medical Institutions, Baltimore, MD USA

**Keywords:** Diabetes, Older adults, Geriatric, Elderly, Glycemic control, Age effects, Comparative effectiveness

## Abstract

**Purpose of Review:**

The purpose of this review is to summarize the current data for comparative effectiveness of glycemic control in older adults.

**Recent Findings:**

In the last several years, professional societies have released guidelines for glycemic control in older adults, generally recommending individualized HbA1c goals. However, recent observational studies demonstrate that many older adults remain aggressively managed and are at increased risk of hypoglycemia. Large randomized trials of older adults with diabetes have failed to show convincing cardiovascular benefit from intensive glycemic control and suggest some microvascular benefit. Additionally, a few studies suggest that suboptimal glycemic control can increase the risk for geriatric syndromes. Emerging research suggests similar safety and efficacy of glucose-lowering therapies in older versus younger adults.

**Summary:**

Overall, there is a paucity of data supporting the benefit of intensive glycemic control in older adults. More research is needed in this vulnerable population.

## Introduction

Optimal glycemic control is often the focus for health care providers when caring for patients with diabetes. However, data has emerged challenging the benefits of tight glycemic control in older adults. Concerns surrounding hypoglycemia and early cardiovascular death with aggressive glucose lowering suggest that aggressive glucose control may cause harm in this population. Currently, the American Diabetes Association (ADA) recommends that for adults ≥65 years old, glycemic goals should be individualized. If cognitively intact and with predicted long life expectancy, hemoglobin A1c (HbA1c) targets should be <7.5%. However, with more complicated medical issues, impaired cognition, and/or impaired physical function, HbA1c targets of <8.0 or <8.5% are recommended, though hyperglycemia leading to symptoms or acute complications should be avoided [1]. The American Geriatrics Society also recommends individualized goals for those ≥65 years old, suggesting an HbA1c target of 7.5–8% overall, 7–7.5% if few comorbidities and good functional status, and 8–9% if poor health and limited life expectancy [2]. Table 1 describes glycemic recommendations for older adults from several organizations. Much of these recommendations are based on expert opinions and observational trials. Randomized trials in older adults are lacking and almost two thirds of recent diabetes trials have been shown to exclude older adults [6], making it difficult to draw conclusions for this population. Therefore, we aimed to review the current evidence for efficacy of glycemic control in older adults.

**Table 1:** Current professional society guidelines for glycemic control in older adults with diabetes

Professional society	Older age definition	Recommendations		
American Diabetes Association (ADA) [1]	≥65 years	Health status	HbA1c	FPG/PPG
		Healthy	<7.5%	90–130 mg/dl
		Intermediate	<8.0%	90–150 mg/dl
		Poor	<8.5%	100–180 mg/dl
American Geriatrics Society [2]	≥65 years	Category	HbA1c	
		Overall	7.5–8.0%	
		Healthy/few comorbidities	7.0–7.5%	
		Poor health	8.0–9.0%	
American Association of Clinical Endocrinologists (AACE) [3]	No age indicated	Category “Less healthy”	Glycemic control “Less stringent”	
International Diabetes Federation [4]	≥70 years	Category	HbA1c	
		Functionally independent	7.0–7.5%	
		Functionally dependent	7.0–8.0%	
		Frail/dementia	Up to 8.5%	
		End of life	“Avoid symptomatic hyperglycemia”	
European Association for the Study of Diabetes [5]	No age indicated	Personalize HbA1c targets based on expected life duration, age, etc.		

Note that ADA has specific guidelines for those in long-term care or skilled-nursing facilities, not listed here

## Current Practice

Despite professional society recommendations, it appears that many older patients remain under strict glycemic control. Analysis of a private insurance and Medicare database from 2006 to 2013, including almost 1.7 million adults , among adults with diabetes ≥65, more than 55% had an HbA1c <7%. The study found that use of insulin in age groups of 65–74 years and ≥75 years increased over the course of the study, reaching over 20% in both groups. The overall rate of hypoglycemia did not decrease over this time period, despite now societal guidelines emerging. A 2016 European cross-sectional study of people with diabetes found that HbA1c level in participants ≥65 were similar to those <65 years old (mean A1c of 7.1%), and that significantly more patients with heart disease had HbA1c values <7% compared to those without [7]. However, strict glycemic control may be linked to unnecessary harm and adverse outcomes in older adults.

## Mortality

The effect of glycemic control on mortality has been explored in observational trials. Assessing the impact of glycemic control, an Israeli group studied 2994 patients ≥65 with new-onset diabetes. After 7 years of follow-up, when compared to the reference group (HbA1c 6.5–6.9%), the group with HbA1c ≥7.5% had a 40% increased overall mortality rate (HR 1.4; 95% CI 1.1–1.6) in a model adjusted for age, sex, smoking, and diabetes medication, among others. There were trends towards higher mortality in both the HbA1c <6.5% and 7–7.4% groups, though these were not significant in the adjusted models [8]. A recent analysis of the National Health and Nutrition Examination Survey (NHANES) study from 1988–2011 studied over 7000 adults ≥65 and found that among those with diabetes, those with an HbA1c ≥8% had a higher risk of all-cause mortality compared to those with an HbA1c <6.5% (HR 1.6; 95% CI 1.02, 2.6) [9]. The Diabetes and Aging Trial was a retrospective cohort study of 71,000 adults ≥60 with diabetes and found a *U*-shaped distribution in rates of mortality by HbA1c. Compared to a group with HbA1c <6%, those with HbA1c 6–9% had a lower mortality risk (for example, HR 0.83; 95% CI 0.76–0.90 for HbA1c 7.0–7.9%), but those with HbA1c ≥11% had an elevated risk (HR 1.31; 95% CI 1.09–1.57) [10].

While observational studies can give insights, large randomized controlled trials allow for more clinically meaningful inferences. The UK Prospective Diabetes Study (UKPDS) trial studied 3867 adults with new-onset diabetes (median 54 years; IQR 48–60 years) randomized to intensive control versus standard control (achieved HbA1c of 7.0 versus 7.9%, respectively) with either sulfonylurea or metformin, over 10 years. Though there was no difference in mortality at the end of the initial study [11], after 10 years of post-trial monitoring, the intensive therapy sulfonylurea group had a 13% risk reduction for all-cause mortality (RR 0.87; 95%CI 0.79–0.96), and the intensive therapy metformin group had a 27% reduction (RR 0.73; 95%CI 0.59–0.89) [12]. The exclusion of older patients (>65 years) at study enrollment makes it difficult to extrapolate to older patients with diabetes. However, it should be considered that at the start of the extension trial, the median age had risen to 62 years of age and thus these more recent findings may be more relevant to older adults.

The Veterans Affairs Diabetes Trial (VADT) [13] (mean age 60 years) and Action in Diabetes and Vascular Disease: Preterax and Diamicron MR Controlled Evaluation (ADVANCE) [14] (mean age 66 years) trials both assessed the effect of intensive glucose control (6.9 versus 8.4% in VADT and 6.5 versus 7.3% in ADVANCE) in an adult type 2 diabetes (DM2) population with pre-existing diabetes (mean 11.5 years in VADT and 8 years in ADVANCE), neither finding any mortality benefit from intensive glucose control even after extension trials of up to 10 years from completion of the original trials [15, 16]. The ADVANCE trial did not demonstrate any differences between older and younger age groups for the primary outcome [14]. The VADT trial did not explore differences in major cardiovascular events or death by age [13].

Similarly, the Action to Control Cardiovascular Risk in Diabetes (ACCORD) trial randomized 10,251 people with pre-existing DM2 (median 10 year duration; mean 62 years), all at high risk for cardiovascular disease, to very intense glycemic targets (achieved A1c 6.4 versus 7.5%). Unexpectedly, the overall study was stopped early (after a mean follow-up of 3.7 years) because of increased all-cause mortality (HR 1.22; 95%CI 1.01; 1.46) in the intensive therapy group [17]. To assess the results in an older population, a subsequent study re-analyzed the data, investigating the modifying effect of age on outcomes, stratifying to age <65 and ≥65 years. The study found that while the overall mortality was increased in the younger group (HR 1.42; 95% CI 1.10; 1.84), there was no statistically increased mortality in those ≥65 [18]. However, more hypoglycemia occurred in the older group.

Overall, observational data suggests that poor glycemic control is associated with higher rates of mortality. Though the age range includes a mix of both middle-aged and older participants, data from large, randomized trials appears to show that intensive control has a neutral effect on all-cause mortality. From the data available, no increased mortality risk has been seen in a population ≥65 with intensive therapy in subgroup analyses.

## Cardiovascular Outcomes

Cardiovascular outcomes are also important to consider. In the UKPDS extension trial, the rates of myocardial infarction were lower in the intensive sulfonylurea (RR 0.85; 95% CI 0.74; 0.97) and metformin (RR 0.67; 95% CI 0.51–0.89) groups, though no significant difference was seen for risk of stroke or peripheral vascular disease [12].

In the ADVANCE extension trial, no difference in macrovascular outcomes were identified [16]. In the original VADT trial, no significant difference in the rates of major cardiovascular events was observed [13]. However, a 5-year follow-up study showed a significantly decreased hazard ratio for the composite cardiovascular endpoint (HR 0.83; 95% CI 0.70–0.99), defined as heart attack, stroke, new or worsening congestive heart failure, amputation for ischemic gangrene, or cardiovascular-related death. This represented 8.6 prevented events per 1000 person-years [15].

In the ACCORD trial, the intensive therapy group had an increase in cardiovascular mortality (HR 1.35; 95% CI 1.04; 1.76), but lower rates of nonfatal myocardial infarction (HR 0.76; 95% CI 0.62; 0.92). There were no differences in the rates of nonfatal stroke or congestive heart failure [17]. The age-specific re-analysis of the ACCORD trial found no increase in cardiovascular mortality in the older group with intensive therapy, while the younger group did have increased risk (HR = 1.71; 95% CI 1.17–2.50), significantly different from the older group (*p* value for interaction = 0.03). However, the younger intensive therapy group had significantly fewer nonfatal MIs than that in the standard therapy group. Though this was not observed in the older population, the interaction *p* values between age groups was not significant for this nor for rates of stroke and congestive heart failure (CHF). It should be noted that the intensive versus standard therapy group had similar percent increases in the rates of severe hypoglycemia in both age ranges, though the older group had higher absolute rates [18].

In 2008, the United States Food and Drug Administration (FDA) issued industry guidance for assessing cardiovascular risk of new diabetes medications which has potential implications for use of these medications in older adults [19]. Consequently, trials since then have investigated the impact of new diabetes medications on composite cardiovascular endpoints. Table 2 describes the cardiovascular risk profiles of common diabetes medications and differences in effect by age, if studied, assessing the comparative effectiveness of these medications in older adults. Both medications studied prior to and after the guidance are included, with medications prior to the guidance sometimes lacking large, randomized trials assessing cardiovascular risk. Comparator groups also vary in these studies, making extrapolation of cardiovascular risk more difficult in older ages.

**Table 2:** Cardiovascular outcomes for diabetes medications in those with diabetes and differences by age

Medication class	Specific medication	Trial	Overall CV events	Differences in CV events by age
DPP-IV inhibitors	*Alogliptin* [20–22, 23••]	EXAMINE	Neutral^a^	No difference^e^
	*Saxagliptin*	SAVOR	Neutral^a^	No difference^f^
	*Sitagliptin*	TECOS	Neutral^a^	No difference^e,f^
GLP-1 agonists	*Lixisenatide* [24–29]	ELIXA	Neutral^a^	No difference^e^
	*Liraglutide*	LEADER	Benefit^a^	Possible neutral effect in adults >=60 years^p^
	*Semaglutide*	SUSTAIN	Benefit^a^	No difference^e^
	*Exenatide ER*	EXSCEL	TBD^g^	
	*Albiglutide*	HARMONY outcomes	TBD^g^	
	*Dulaglutide*	REWIND	TBD^g^	
SGLT-2 inhibitors	*Empagliflozin* [30••, 31, 32]	EMPA-REG	Benefit^a^	Benefit if ≥65 years, neutral if <65 years (*p* = 0.01 for interaction)
	*Canagliflozin* [33]	CANVAS	Benefit^a^	Possibly greater benefit in adults >=65 years old^p^
	*Dapagliflozin*	DECLARE	TBD^g^	
Insulin	Insulin glargine [34]	ORIGIN	Neutral^a^	No difference^e^
Sulfonylureas (SUs)/meglitinides	All SUs [35, 36]		? Harm^b^	Not studied
	2nd generation		Neutral^b^	Not studied
Thiazolidinediones	Rosiglitazone [37–40]		? Harm^b^	Not studied
	Pioglitazone		Neutral^c^	Not studied
Biguanides	Metformin [12, 41]		Benefit	Not studied
Alpha-glucosidase inhibitors	Acarbose [42]	ACE	TBD^g,h^	TBD
Amylin agonists	Pramlintide [43]		Neutral^a^	Not studied

Medications in italics were subject to the 2008 FDA’s industry guidance for assessing cardiovascular risks

^a^Outcome is major adverse cardiovascular event (MACE): cardiovascular death, nonfatal myocardial infarction, or nonfatal CVA

^b^Based off meta-analyses of cardiovascular outcomes; no large trials

^c^Based on cardiovascular outcomes, but not MACE

^d^When stratified by <60 and ≥60 years

^e^When stratified by <65 and ≥65 years

^f^When stratified by <75 and ≥75 years

^g^Trial ongoing

^h^Trial studying both those with prediabetes and DM2

^p^ value for interaction by age was not significant

Regarding head-to-head medication comparisons, the A Diabetes Outcome Progression Trial (ADOPT) trial studied metformin, glyburide, and rosiglitazone as monotherapy in adults with new-onset type 2 diabetes. While the primary outcome was time to monotherapy failure, cardiovascular endpoints were also evaluated. Rates for overall and cardiovascular deaths did not differ, but compared to the glyburide group, those taking rosiglitazone were more likely to have CHF events (HR 2.20; 95% CI 1.01–4.79). No statistical difference was observed between metformin and glyburide [44]. A meta-analysis of randomized and observational trials found that CHF adverse events were less common with metformin versus sulfonylureas (HR 0.7–0.85, moderate evidence) and more common in TZDs versus sulfonylureas (OR 1.68, moderate evidence) [45]. Another meta-analysis found lower risk of cardiovascular mortality in those using metformin monotherapy versus sulfonylurea monotherapy in both randomized (RR 0.6–0.7) and observational studies (HR 0.6–0.9) [46].

Two meta-analyses from 2011, each with about 30,000 adults with diabetes (not restricted to, but including older adults), saw no reduction in all-cause mortality with intensive glycemic control (defined as goal HbA1c <6–7%, depending on the trial) [47, 48]. A reduction in nonfatal myocardial infarction was observed (RR 0.85; 95% CI 0.74–0.96), but also noted a two-fold higher rates of severe hypoglycemia [47].

Overall, risk for cardiovascular disease appears to increase with poorer control. Those with new-onset diabetes and in middle-ages appear to have few MIs with tighter control, but in those with longer-duration diabetes and in older adults, the data for benefit is mixed.

## Microvascular Outcomes

Regarding microvascular outcomes, at the end of the UKPDS trial, the intensive therapy group had a 25% reduction (RR 0.75; 95% CI 0.60–0.93) in microvascular disease, defined as renal failure, death from renal failure, retinal photocoagulation, or vitreous hemorrhage [11]. At the end of the extension study, with almost 17 years of total follow-up, the intensive therapy group on sulfonylureas saw a persistent 24% reduction in microvascular disease (RR 0.76; 95% CI 0.64; 0.89), but this did not persist in the metformin group (RR 0.84; 95% CI 0.60; 1.17) [12].

The original VADT trial showed a reduction of overall progression of albuminuria in the intensive group (9.1 versus 13.8%; *p* = 0.01), but no difference in new onset or progression of neuropathy or retinopathy [13]. Post-trial microvascular observational data for VADT is not yet available.

In the ADVANCE trial, the intensive group saw a 14% reduction in major microvascular events (HR 0.86; 95% CI 0.77–0.97), which appeared driven by a reduction in nephropathy, as there was no significant difference in retinopathy rates [14]. During the extension study and after an additional 5.4 years, the reduction in microvascular events was lost (again with no benefit in rates of retinopathy), though the rates of end stage renal disease were 46% lower in the intensive therapy group (HR 0.54; 95% CI 0.34–0.85). However, it should be noted that there were very few events overall [16].

In the ACCORD study, the intensive control group had significantly lower rates of incident microalbuminuria and macroalbuminuria, less surgery for cataracts, and improvement in certain measures of neuropathy, but the composite microvascular outcomes were not significantly different between groups [17, 49]. A subgroup of 2856 took part in the ACCORD Eye study, assessing for the progression of retinopathy over the 4 years of follow-up. At the end of the study, the intensive control group had a 40% lower risk of progression (OR 0.60; 95% CI 0.42; 0.87) [50].

Overall, data for microvascular benefits of intensive control in trials suggest that older adults may have some benefits. More benefit is observed in those with new-onset diabetes, but even for those with longer-duration diabetes in older populations, there is a benefit of more intensive glucose control on diabetic kidney disease and/or retinopathy, though data is mixed.

### Geriatric Syndromes

Geriatric syndromes are health conditions commonly occurring in older adults that do not fit into discrete disease categories [51]. Though diverse, these syndromes share several common features: they are highly prevalent among older adults and are associated with poor outcomes in quality of life, disability, and morbidity [52, 53]. Another feature is the multifactorial origin of these syndromes. Unlike traditional medical syndromes driven by a single underlying factor, geriatric syndromes result from a number of interacting intrinsic, external, and iatrogenic factors [54, 55]. The complex roots of these syndromes can pose significant challenges for clinicians and researchers alike in elucidating their etiology, natural history, presentation, and outcomes. The following sections will discuss the impact of glycemic control in older adults on urinary incontinence, polypharmacy, cognitive dysfunction, functional decline, falls, fractures, and frailty.

#### Incontinence

A limited number of observational studies in older adults have explored the relationship between glycemic control and incontinence. In a large survey of Australian men and women (mean age 60 years), adults with diabetes were nearly three times more likely (OR 2.74; 95% CI 1.40–5.37) to experience fecal incontinence than nondiabetics [56]. In the US National Health and Nutrition Examination Survey, women with HbA1c of 6.5–8.5% had a 13% (95% CI, 1.03–1.25) increased risk for any urinary incontinence and a 34% (95% CI 1.06–1.69) increased risk for stress incontinence for each one-unit increase in HbA1c [57]. However, this study included a wide age range (ages 20–85) and the mean age was 61.9 ± 17 years [57]. Conversely, in a study of Dutch women (mean 59 years), HbA1c level was not associated with incontinence [58].

#### Polypharmacy

Approximately 35–39% of US noninstitutionalized adults ≥65 years take ≥5 prescription medications; [59, 60] significantly increased from about 13% in the late 1980s [60]. Regarding risk of hypoglycemia, Noale et al. found a significant association between older adults with diabetes (mean age 73 years) experiencing hypoglycemic events and taking at least five prescription medications [61].

#### Cognitive Dysfunction

Increasing attention has focused on the effects of glycemic control on cognitive decline and incidence of dementia in observational studies. To explore cognitive outcomes, a Chinese cross-sectional study of adults ≥65 years with diabetes and mild cognitive impairment found significant associations with longer duration of diabetes (OR 1.33), higher HbA1c (OR 1.25), and higher fasting glucose (OR 1.17) [62]. Similarly, a group using data from the Israel Diabetes and Cognitive Decline study of community-dwelling older adults with DM2 found significantly poorer cognitive performance with higher HbA1c in categories of executive functioning, semantic categorization, attention/working memory, and overall cognition, though not for episodic memory [63]. Another registry study of older adults with DM2 (mean 73 years) over mean 8.7 years of follow-up studied how HbA1c trends related to cognitive decline. They found that those with high HbA1c at baseline with decreasing values over time had the worst cognitive performance, followed by those with moderately high baseline or increasing HbA1c over time. Those with the lowest baseline HbA1c and with stable HbA1c performed best on cognitive testing [64].

When specifically investigating brain volumes, a Japanese study of adults ≥65 years with diabetes found that longer duration of diabetes and higher 2-h glucose value on oral glucose tolerance testing were associated with lower total brain to total intracranial volume and particularly with lower hippocampal volume ratios, suggesting higher degrees of brain atrophy [65]. A similar pattern was seen in another study showing higher degrees of atrophy in older adults with DM2, particularly in those with longer diabetes duration and higher fasting glucose levels [66].

There have been few randomized controlled trials that have explored the effects of intensive glucose control on cognitive outcomes. The Memory in Diabetes (MIND) trial (mean 63 years) was a substudy of the ACCORD trial in which a subset of patients had Digit Symbol Substitution Test (DSST) and brain MRIs at baseline and 40 months. The intensive control group had significantly higher total brain volumes at 40 months, but there was no significant difference in DSST scores [67].

To more specifically address the effect of hypoglycemia on cognitive status, a prospective study of a subset of the Health, Aging, and Body Composition (Health ABC) study of community-dwelling older adults investigated whether those with diabetes and a hospital visit for hypoglycemia had higher rates of incident dementia. Those with at least one episode of hypoglycemia were at two-fold higher risk of future dementia (34.4 versus 17.6%, *p* < .001) [68]. A Korean prospective observational study had similar findings, with a linear trend of increased dementia risk with increasing number of hypoglycemic events during a follow-up of about 3.5 years. After adjustment for medical history and medications, those with one or more episodes of hypoglycemia had a 2.7 times higher risk of dementia (HR 2.69; 95% CI 1.08; 6.69), though no significant trend was found for cognitive dysfunction without dementia [69].

Regarding the effect of dementia on risk of hypoglycemia, those with dementia appear to be at higher risk of hypoglycemia. The Health ABC trial previously described noted those with dementia had higher rates of hypoglycemia (14.2 versus 6.3%, *p* < .001) [68]. Though an assumption might be that those with dementia are given less aggressive glycemic goals, this is not always the case: a retrospective Veterans Affair study of adults ≥65 years old with DM2 and dementia showed that 52% had an HbA1c <7%. The older age categories appeared to have higher likelihood of tighter control, with odds ratios of 1.16 and 1.13 in the 75–84 and ≥85 groups, respectively [70]. A study of adults with DM2 using a German/Austrian database also illustrated this, showing that in those using insulin or sulfonylureas, the rates of severe hypoglycemia, defined as requiring assistance from others to remedy, (14.8 ± 0.6 versus 10.4 ± 0.2 events per 100 patient-years, *p* < 0.001) and hypoglycemia with coma (7.6 ± 0.4 versus 3.9 ± 0.1 events per 100 patient-years, *p* < 0.001) were significantly higher in patients with dementia, much of which was vascular dementia in that study [71]. Post-hoc analysis of the ACCORD trial data studied the predictive effect of poor cognitive function on the outcome of hypoglycemia. They found that at just over 3 years of follow-up, a 5 point lower score on the DSST was predictive of first episode of hypoglycemia requiring medical assistance (HR 1.13; 95% CI 1.08–1.18). A subsequent round of cognitive testing 20 months later found that cognitive decline increased the risk of hypoglycemia the most in those with the lowest baseline cognitive testing scores, with no effect of randomization to intensive versus standard glycemic strategy [72].

Overall, it appears that there is evidence from observational studies to support that either long-standing hyperglycemia or episodes of severe hypoglycemia increase the risk for cognitive decline and dementia. Those already with dementia are at higher risk for hypoglycemia and may benefit from having relaxed glycemic goals. There is a paucity of randomized clinical trials investigating the effects of intensive glucose control on cognitive decline in persons with diabetes.

#### Functional Decline

Older adults with poor glycemic control are likely at greater risk for frailty and functional outcomes [73]. Declines in physical function are common among adults ≥65 years [74, 75] and have been shown to predict disability and death [76, 77].

Older adults with higher blood glucose levels are likely at greater risk for functional declines [73], though findings have been mixed. In a study of 5035 US community-dwelling adults (mean age 75 years), those with diagnosed diabetes and poor glycemic control (defined as HbA1c >7.0%) had a significantly greater prevalence of functional disability in crude and adjusted (for demographics, health behaviors, and comorbidities) models [78]. Among US community-dwelling women aged 70–79 at baseline, HbA1c ≥8.0% (compared to <5.5%) was associated with an increased risk of developing walking difficulty (HR = 3.47, 1.26–9.55) low walking speed (HR = 2.82, 1.19–6.71), and low physical performance (HR = 3.60, 1.52–8.53) during an average follow-up of 8+ years [79]. Significant associations have also been found between elevated HbA1c levels and lower lean body mass, as well as lower muscle strength [80, 81]. In a nationally representative study of US adults aged 60 or older, those with diabetes had 2–3 times greater probability of disability across functional groups, including lower-extremity mobility, activities of daily living (ADL), and instrumental activities of daily living (IADL) disability [82]. However, in further analyses that adjusted for demographics and comorbidities, poor glycemic control (HbA1c ≥8%) was not significantly associated with disability in these functional groups [82]. Among US nursing home eligible adults (mean 80 years), older adults with HbA1c 8–8.9% had a significantly lower risk of functional decline or death at 2 years, when compared to those with HbA1c 7–7.9% [83]. Additionally, a study among Italian nursing home residents found that ADL declines were significantly associated with tighter glycemic control and hypoglycemia [84].

The Look AHEAD (Action for Health in Diabetes) study was a multicenter, randomized, controlled trial which enrolled overweight or obese persons with type 2 diabetes designed to determine whether intentional weight loss would reduce morbidity and mortality. A substudy (mean age 59 years) analyzed the effect of intensive lifestyle intervention on functional decline. At year 4, the lifestyle intervention group had a relative reduction of 48% in the risk of loss of mobility (OR 0.52; 95% CI 044; 0.63), with weight loss and improved fitness serving as mediators [85]. Similarly, the Diabetes Prevention Program trial randomized adults (mean age 51 years) with prediabetes to metformin, placebo, or intensive lifestyle modification (with the goals of 7% weight loss and 150 min of physical activity per week). At 3 years of follow-up, those in the intensive lifestyle modification group showed improvement in physical function, bodily pain, and vitality scores compared to the other groups [86].

#### Falls

Approximately one third of community-dwelling adults aged 65 and older experience a fall each year worldwide [87], with estimates up to 50% annually with increased age and institutionalization [87, 88]. Falls are a leading cause of injury-related deaths among adults ages 65 and older in the USA [89], and up to 20% of falls among older adults results in serious health outcomes [90].

Regarding the association of glycemic control and falls, a retrospective study of adults ≥75 with diabetes, tighter glycemic control (HbA1c ≤ 7) was associated with a 32% greater risk of falls compared to those with HbA1c >7 [91]. A separate retrospective analysis among nursing home residents found that prevalence of falls was greater among those with HbA1c <7 and >9% across age groups 65–74, 75–84, and 85+ years old [92]. A study of older Japanese adults (mean age 76 years) with diabetes found that the presence of hypoglycemia was a significant risk factor for falls [93]. Alternately, a Taiwanese study of older adults (mean 76 years) found that hyperglycemia was one of several factors associated with greater probability of falling [94]. However, hyperglycemia is not universally associated with increased fall risk.

A subset of the ACCORD trial investigated the incidence of nonspine fractures (including hip, ankle, and forearm fractures) in 7287 participants from the original study (mean 63 years), comparing the standard versus intensive control groups, showing no significant difference in the incidence of nonspine fracture or falls [95].

#### Fractures

Compared to younger persons, older adults account for a large proportion of fractures each year, most often a result of falling [96]. Hospitalizations for hip fractures in older adults are estimated at more than 300,000 per year [97], and mortality rates 1 year after hip fracture have been reported at over 25% in observational studies [98].

A large prospective study of US adults aged 65 and older with diabetes mellitus found that those with baseline HbA1c 6.5–6.9% were at the lowest risk for fracture over an average 3.3 years of follow-up [99]. In a study of Taiwanese older adults (ages ≥65) with diabetes, incident hip fracture rates were the lowest among those with HbA1c 6–7%. Over an average 7.4 years of follow-up, those with HbA1c levels of ≥9% were at the highest risk for hip fracture [100]. Conversely, a case-control study among older Chinese hip fracture patients found that those with HbA1c <6 and 6.1–7% had greater odds of hip fracture compared to patients with HbA1c >8% [101]. A Dutch study of older adults (mean age 69 years) found that those with baseline HbA1c ≥7.5% were at much greater risk for fracture (hip and/or wrist) over an average 12 years of follow-up compared to participants with no diabetes or with HbA1c <7.5% [102]. In a large database of Medicare patients (≥65) with diabetes, those with hypoglycemic events during a 1-year baseline period had a significantly higher odds of fall-related fractures compared to those without hypoglycemic events [103]. Randomized trials are lacking, but in the substudy of the ACCORD trial reference above, no significant difference was observed in the incidence of nonspine fracture between intensive and standard control groups [95].

#### Frailty

Frailty is conceptualized as an increased vulnerability to develop adverse health outcomes when encountering a stressor [104]. Approximately 15% of older adults in the USA are frail; [81] worldwide estimates vary [105]. It is most commonly assessed using the physical frailty phenotype [106], which includes five criteria: weight loss, weakness, slowness, exhaustion, and low activity [107, 108].

Older adults with diabetes and/or hyperglycemia are at increased risk for frailty [73]. A population-based prospective study of US adults ≥65 explored the incidence of frailty over a mean 4.8 years of follow-up [109]. The incidence of frailty was 37% in older adults with diabetes during follow-up, compared to 30.4% incidence in those without diabetes (HR: 1.52, 95% CI 1.19–1.94) [109]. In a Spanish cohort of adults aged ≥60 at baseline, the incidence of frailty during 3.5 years of follow-up was 11.3% for older adults with diabetes, compared to 5.4% among nondiabetic older adults [110]. Among those with diabetes, HbA1c levels greater *or* lesser than the median average (7.6%) were associated with increased risk for developing frailty [109]. In a prospective observational study of US community-dwelling women enrolled at ages 70–79, baseline HbA1c ≥8.0% (compared to <5.5%) was associated with a three-fold increased risk of developing frailty over a mean 8+ years of follow-up [79].

## Other Considerations in Establishing Glucose Targets in Older Adults with Diabetes

Avoiding both hypoglycemia and hyperglycemia is important in the care of older adults with diabetes. The Diabetes and Aging study showed that for those with short (0–9 years) or long-duration(≥10 years) of diabetes, rates of hypoglycemia were much more common in the oldest age group, with double the risk of hypoglycemia in those ≥80 versus those 60–69. Across all age categories, those with long-duration diabetes had about three times more hypoglycemia events than those with short-duration [111]. Using another US database, the frequency of admission for hypoglycemia was 0.59 per 1000 person-years in those ≥65 years old and only 0.16 in those <65 years old. Older age and sulfonylurea or insulin use were all risk factors for hypoglycemia admissions. Older patients using both insulin and sulfonylureas were at the highest risk, with an odds ratio of 4.7 (95% CI 3.7–6.1) for hypoglycemia [112]. Similarly, in the Outcome Reduction with an Initial Glargine Intervention (ORIGIN trial), participants on sulfonylureas at baseline (mean age 64 years) and those allocated to initiating insulin glargine were at higher risk of hypoglycemic events [113]. Hypoglycemia is potentially dangerous, as evident by a Medicare database study from 1999 to 2011 which found that the 30-day mortality rate after admission for hypoglycemia was 19.9% [114••].

Avoiding uncontrolled hyperglycemia is also of importance. A case-control study from Taiwan examined the impact of admission for hyperglycemic crisis on mortality rates. They found that compared to controls, those with one episode of hyperglycemic crisis had mortality hazard ratios of 2.85 (95% CI 2.60–3.12), increasing to 4.53 (95% CI 3.36–6.09) with more 2+ episodes, even after adjusting for demographic information. The mortality rate was highest the first month after admission and remained elevated for up to 6 years [115]. Similarly, a Medicare database study found that the 30-day mortality rate after admission for hyperglycemia was 17.1% [114••].

## Nursing Home Residents

To specifically address those in a nursing home population, a retrospective cross-sectional study of 583 adults (mean age 79) with DM2 living in a long-term care facility for ≥3 months found that the rates of hypoglycemic episodes increased with age, but were lowest in those with HbA1c >9. In all age categories, the risk of falls followed a *U*-shaped curve, with the highest rates in those with HbA1c <7 and >9%. The overall rates of hospitalization for glycemic issues were low [92]. Another, longitudinal cohort study of community-dwelling, nursing home eligible older adults with diabetes (mean age 80) assessed the impact of HbA1c on death and functional decline (of which 75% of the population experienced these outcomes in the 2 years of follow-up). The study noted that high HbA1c was associated with significantly lower rates of death or functional decline at 2 years. When adjusting for demographics and comorbid conditions, they found that an HbA1c of 8–8.9% was associated with a lower likelihood of death or functional decline (HR 0.88; 95% CI 0.79; 0.99) than an HbA1c 7–7.9%. Though it did not meet significance, those with HbA1c <7% had the highest risk of death or functional decline, while HbA1c ≥9% had a nonsignificantly risk reduction compared to the reference [83]. A Veterans Affair cohort of nursing home residents ages ≥65 (mean 76 years) with diabetes found no significant association between HbA1c level and functional decline or death during 24 months of follow-up, even for those with HbA1c >9%. The lack of association persisted in subgroups based on medication usage. Admittedly, the length of nursing home stay was short (<6 months), but the results were similar when restricted to residents of >6 months [116].

Overall, observational studies suggest that those in a nursing home setting had similar, if not better, outcomes with a relatively higher HbA1c level.

## Conclusion

The numbers of older adults with diabetes is expected to increase dramatically in the next decades [117], and appropriately managing diabetes in this population will become ever more important. At this point, data for improved outcomes with intensive glycemic control is mixed. Though some observational studies suggest reduced mortality from lower HbA1c levels, randomized trials have not necessarily demonstrated benefits of aggressive glucose lowering in older adults. Microvascular outcome data demonstrates no definitive overall improvement in older adults with tighter control, though benefits in retinopathy and diabetic kidney disease have been observed in RCTs. Both hyperglycemia and hypoglycemia are related to an increased risk of geriatric syndromes such as cognitive dysfunction, falls, fractures, and functional disability in most observational studies. Taken together, the current recommendations for individualized treatment goals in older adults by many professional societies are supported by the evidence to date. However, further large, randomized trials involving older adults with diabetes are needed to better understand the comparative effectiveness of glycemic control in the future.
